# Scientific opportunities in resilience research for cardiovascular health and wellness. Report from a National Heart, Lung, and Blood Institute workshop

**DOI:** 10.1096/fj.202201407R

**Published:** 2022-12

**Authors:** Herman A. Taylor, Toren Finkel, Yunling Gao, Scott W. Ballinger, Rebecca Campo, Rong Chen, Shu Hui Chen, Karina Davidson, M. Luisa Iruela-Arispe, Cashell Jaquish, Nathan K. LeBrasseur, Michelle C. Odden, George J. Papanicolaou, Martin Picard, Pothur Srinivas, Olga Tjurmina, Michael Wolz, Zorina S. Galis

**Affiliations:** 1 Cardiovascular Research Institute Morehouse School of Medicine, Atlanta, Georgia, USA; 2 Morehouse-Emory Cardiovascular Center for Health Equity, Atlanta, Georgia, USA; 3 Harvard Chan School of Public Health, Atlanta, Georgia, USA; 4 Emory School of Medicine, Atlanta, Georgia, USA; 5 University of Pittsburgh School of Medicine, Pittsburgh, Pennsylvania, USA; 6 Division of Cardiovascular Sciences, National Heart, Lung, and Blood Institute, National Institutes of Health, Bethesda, Maryland, USA; 7 University of Alabama Heersink School of Medicine, Birmingham, Alabama, USA; 8 Icahn School of Medicine at Mount Sinai, New York, New York, USA; 9 Sema4, Stamford, Connecticut, USA; 10 Feinstein Institutes for Medical Research, Northwell Health, New York, New York, USA; 11 Feinberg School of Medicine, Northwestern University, Chicago, Illinois, USA; 12 Mayo Clinic, Rochester, Minnesota, USA; 13 Stanford University, Stanford, California, USA; 14 Columbia University Irving Medical Center, New York, New York, USA; 15 New York State Psychiatric Institute, New York, New York, USA

**Keywords:** adaptation, aging, cardiovascular disease, cardiovascular health, genetic plasticity, health disparities, homeostasis, resilience, stress

## Abstract

Exposure of biological systems to acute or chronic insults triggers a host of molecular and physiological responses to either tolerate, adapt, or fully restore homeostasis; these responses constitute the hallmarks of resilience. Given the many facets, dimensions, and discipline-specific focus, gaining a shared understanding of “resilience” has been identified as a priority for supporting advances in cardiovascular health. This report is based on the working definition: “*Resilience is the ability of living systems to successfully maintain or return to homeostasis in response to physical, molecular, individual, social, societal, or environmental stressors or challenges*,” developed after considering many factors contributing to cardiovascular resilience through deliberations of multidisciplinary experts convened by the National Heart, Lung, and Blood Institute during a workshop entitled: “*Enhancing Resilience for Cardiovascular Health and Wellness.”* Some of the main emerging themes that support the possibility of enhancing resilience for cardiovascular health include optimal energy management and substrate diversity, a robust immune system that safeguards tissue homeostasis, and social and community support. The report also highlights existing research challenges, along with immediate and long-term opportunities for resilience research. Certain immediate opportunities identified are based on leveraging existing high-dimensional data from longitudinal clinical studies to identify vascular resilience measures, create a ‘resilience index,’ and adopt a life-course approach. Long-term opportunities include developing quantitative cell/organ/system/community models to identify resilience factors and mechanisms at these various levels, designing experimental and clinical interventions that specifically assess resilience, adopting global sharing of resilience-related data, and cross-domain training of next-generation researchers in this field.

## 1 | INTRODUCTION

The scientific concept of resilience can be traced back to the iconoclastic English scientist and polymath, Robert Hooke. Around the year 1660, Hooke noted that the degree of displacement of an object (e.g., a spring) from its equilibrium point, was proportional to the force applied.^1^ Moreover, under most conditions, the object appeared to return to its original shape and size upon removal of the force. The resulting equations governing these principles, known as Hooke’s Law, provided a roadmap for understanding stress and resilience in the mechanical world. These ideas have remained largely intact over the ensuing 350 years. However, the concepts of stress and resilience extend far beyond simple springs and other mechanical objects. Increasingly, these notions are being applied to biology and human physiology, where the simple linear relationships delineated by Hooke no longer apply. The mission of the National Institutes of Health is to seek fundamental knowledge about the nature and behavior of living systems and the application of that knowledge to enhance health, lengthen life, and reduce illness and disability. Within this context, a multidisciplinary group of experts was convened in a workshop^2^ organized by the National Heart, Lung, and Blood Institute (NHLBI) to review the current state of the science and emerging concepts regarding the resilience of living systems, identify opportunities and barriers, and develop considerations for immediate and longer-term research that could lead to advances in maintaining and returning to cardiovascular health (CVH).

Discussions tackled the various facets and scales where resilience may be observed in living systems, from the sub-cellular to the epidemiological in scale, and evaluated known stresses, challenges, and corresponding responses. The need to reach a shared understanding of the concept of resilience was quickly identified as the key requirement to enabling progress in this multidisciplinary area of research. The participants agreed on an operational definition of resilience as *“the ability of living systems to successfully maintain or return to homeostasis in response to physical, molecular, individual, social, societal, or environmental stressors or challenges*.” Discussions highlighted the significant gaps in knowledge, from the molecular to the societal determinants for heterogeneous responses to stress, for instance, how one cell or tissue type is more resilient in the setting of age or disease, or why, under similar socio-economic stress, many individuals will develop hypertension, yet some will not. The molecular underpinnings of vulnerability in some populations, elderly or diseased, as observed through inability to maintain or return to tissue homeostasis and normal physiology following various stresses, remain unknown. All participants agreed that understanding the basis of resilience at the molecular, cellular, tissue, individual, and population levels are likely to provide a guide for maintaining overall CVH and developing new preventive and restoring strategies useful for a wide array of cardiovascular diseases (CVDs). We share our findings of the challenges and suggestions regarding research opportunities to advance our understanding of what defines and determines CV resilience and how best to translate that knowledge into new preventive and therapeutic approaches. Many conclusions are relevant to the overall relationship between resilience, health, and disease.

## 2 | MOLECULAR BASIS OF RESILIENCE

The human genome holds the blueprint for life. Its molecular manifestation provides an important tool for understanding resilience. Most current genomic studies focus on disease-affected individuals seeking to pinpoint the genetic origin of specific diseases. On the other hand, shifting the focus of genomic and transcriptomic analysis to healthy individuals can help identify resilient cohorts, those who show no signs of disease despite the presence of genetic variants or other challenges that would induce disease in the rest of the population.

Studies searching for disease-resistant individuals have helped uncover naturally occurring protective mechanisms that are important not only for providing fundamental insights into the molecular basis of resilience but also for helping guide the development of novel treatments for people who are affected by those diseases. A stellar example of naturally occurring ‘loss of function’ mutations that turned out to be protective from CVD or promote CVH was the discovery of an association between protection from atherosclerotic disease and genetic variants of proprotein convertase subtilisin/kexin type 9 (PCSK9) that lead to lifelong low blood levels of lipoproteins. This has resulted in the rapid development of effective lipid-lowering therapeutic interventions based on PCSK9 inhibitors that have effects beyond those of traditional statins.^3^

Such integrated approaches could also identify individuals who harbor mutations for known childhood-onset disease but never manifest the disease clinically. Integrative analysis of genomic and electronic medical record (EMR)-based data could provide invaluable information in identifying and characterizing individuals in the general population who exhibit extreme resilience.

The following are potential opportunities to accelerate the process of identifying and studying resilience, especially in genetics: harness big data to establish a large analytic platform, develop effective systems to acquire longitudinal and quantitative clinical phenotyping data through physicians’ notes to complement the molecular genetic information already available, and through high-density data including from mobile App for analysis of genetic data, evaluate RNA-Seq data from individuals exposed to similar stressors but who showed different responses, develop automatic tools to evaluate genetic variant pathogenicity, establish easy access to quality-controlled databases, validate or invalidate resilience hypotheses relevant to human in vitro and in vivo models, and generate new biological questions using these big data.

Understanding the key mechanisms of resistance to disease at the cellular/tissue/organ levels is important to gain insights into the molecules and pathways involved in CV resilience and to differentiate them from pathways involved with vulnerability (predisposition) to pathophysiology. Endothelial dysfunction is central for CVD, thus pathways that preserve endothelial cell resilience could play a key role in CV resilience and warrant further in-depth research.^4^

Genetic variations and unfavorable environments lead to vulnerability. Genetic plasticity, molecular and cellular adaptability lead to cell-autonomous resilience. Resilience is a moving target and a dynamic process. Biological systems have evolved to be resilient to the environment and are able to return to homeostasis through systemic adaptations such as developmental compensations, feedback mechanisms, and redundancy of functions.^5,6^ These interactions of micro- and macro-environments with the organism are essential in regulating cell/tissue/organ resilience. Our understanding of the specifics may be enhanced by searching beyond previous efforts that have been centered on disease-causation rather than disease-resistance.

The divergences in the clinical course of patients with coronavirus disease-2019 (COVID-19) have provided a natural laboratory to study the tremendous impact of resilience in human health. Evidence suggests that age, sex, genetic background, pre-existing condition, and health disparity, all play a role in a patient’s response to the severe acute respiratory syndrome coronavirus 2 (SARS-CoV-2). Recent use of genome-wide association studies (GWAS) and Mendelian randomization have allowed some insights into specific pathways that may define resilience to viral infection.^7^ It remains unclear, however, whether the genetic factors that mediate COVID-19 resilience will extend to other illnesses or scenarios, although recent studies in animal models suggest at least some factors might be shared.^8^ Another important lesson that has emerged from this global health threat was the resilience of the scientific enterprise itself, overcoming the challenging research circumstances and knowledge gaps through engagement in wide international collaborations and dialogue across the entire spectrum of scientific expertise and sectors, open data sharing, and the rapid dissemination and translation of findings into preventive and treatment interventions.

Transcriptional flexibility thresholds (elasticity) offer a measure of resilience at the molecular level. Tightly controlled pathways are not tolerant to changes. For example, both the Vascular Endothelial Growth Factor (VEGF) and Notch signaling pathways, endure alterations poorly. Disruptions in VEGF and Delta-like-4 levels by inactivating a single allele result in embryonic lethality. Similarly, in adult settings experimentally imposed changes in VEGF or Notch signaling result in pathologies.^9,10^ In these pathways, levels of the rate-limiting molecules are critical and to ensure such levels, regulation is present at the transcriptional, translational, and post-translational steps. Thus, flexibility in how individuals maintain adequate functions in these pathways is crucial for their adaptation to stressors. In turn, genes that display more flexible expression exhibit greater degrees of tolerance to disruptions and/or stressors. Genes are dynamic units capable of responding to the smallest perturbations in their environment. Plasticity in the transcriptome offers an important first level for resilience, once this adaptation is lost, cells and tissues experience stress. Transcriptome analysis across individuals may provide a promising approach to identifying the mechanisms promoting transcriptional elasticity and adaptability to environmental stressors.

The biomedical research field needs to further expand to identify genetic, molecular, and signaling pathways that participate in resilience. Computer modeling and high-level bioinformatics will be central to decoding data and building models and hypotheses, predicting outcomes, and interpreting combinatorial effects. Technological advances in omics will most certainly accelerate the identification of relevant gene cohorts and molecular interactions that promote resilience. The creation of “cloud platforms” for data consultation would accelerate scientific inquiry worldwide and leverage discoveries. We must move from an assessment of the effect of a single gene to the analysis of networks of genes that operate together and impose functional reciprocity and feedback. Emerging disease models and organ-on-a-chip technologies could facilitate the understanding of resilience. The assessment of genetic and non-genetic mechanisms that enable adaptation to environmental, chemical, and pathogen challenges should be stimulated. To objectively assess resilience, validated and standardized biological and/or physiological markers and measurements for resilience need to be established. In seeking the molecular mechanisms of resilience that drive adaptation and boundaries of molecular resilience, the reactive oxygen species (ROS), epigenetic modifications, and ligand-activated transcription factors may play critical roles.

## 3 | IMPLICATIONS OF INFLAMMATION RESOLUTION: HOW MODULATING FACTORS CONTRIBUTE TO RESILIENCE

The body’s natural mechanisms that resolve inflammation could provide insight into CV resilience. Classically, inflammation has been separated into two main categories: acute and chronic. Acute inflammation is typically thought of as a process by which the body protects itself from outside invaders such as microbes, viruses, or physical injuries (such as surgical-induced tissue injury) that resolve quickly. While the acute inflammatory response is protective, chronic inflammation is a unifying component of systemic organ diseases including CVD, diabetes, etc. For many years, it was thought that the resolution of the acute inflammatory response was a passive event where the factors involved in inflammation were diluted away from the site of inflammation (such as dissipation of inflammation factors by blood flow), leading to the cessation of leukocyte recruitment. Recent studies have shown that this is not the case, and that resolution of inflammation can be an active process, which has vast implications for CV resilience. An overview of research on the structural elucidation of novel resolution phase mediators and their functions as an agonist of resolution of inflammation was illustrated by Dr. Charles N. Serhan in 2018.^11^

The resolution of acute inflammation gives insights applicable to the treatment of common inflammatory diseases such as CVD.^11–13^ When discussing CVH and resilience, the workshop focused on the resolution of inflammation and how specialized pro-resolving mediators (SPMs) may contribute to CV resilience. Specifically, understanding the role of SPMs, such as the resolvins, lipoxins, protectins, and maresins, their biosynthetic pathways, and pro-resolving receptors in countering inflammation and stimulating resolution, might shed light on how tissue resilience may contribute to CV resilience, cardiac vasculature repair, and an overall increase in CV health span.

Studying how the body naturally resolves inflammation may provide alternative treatments that leverage the body’s natural defenses and elucidate potential therapies for chronic illnesses involving inflammation. For instance, molecules derived from essential polyunsaturated fatty acids (PUFAs) have been shown to be potent mediators of polymorphonuclear leukocytes. In early 2008, it was suggested that anti-inflammatory lipid mediators and molecules that resolve inflammation, such as E-series and D-series resolvins, have a protective impact on the development of atherosclerosis via the anti-inflammation and pro-resolution lipoxygenase pathways.^14^ Advances in understanding the effect of modulating factor pathways in the resolution of inflammation and how they interplay with SPM have been made, but it is unclear how these factors affect CVH. For instance, some studies have suggested that omega-3 fatty acids may protect against vascular inflammation regardless of an individual’s cholesterol and triglyceride levels.^15^ The ethyl ester form of the omega-3 fatty acid eicosapentaenoic acid (EPA) is a substrate of the SPM E-series resolvin, RvE1, thereby suggesting that SPM may work to preserve CVH. Diets rich in n-3 polyunsaturated fatty acid were shown in animal models to positively affect cognition and cerebral vasculature.^16^ Results from these studies suggest that certain fatty acids may improve resilience and aid in the recovery of the blood–brain barrier from trauma. Nonetheless, more basic and clinical evidence needs to be collected in order to understand who might benefit, and how, from these types of treatments.^11,17–20^ Understanding the interplay between chronic inflammation and delayed CV resilience will require deciphering the markers of chronic inflammation. Normal variations in the individual SPM profile in relation to characteristics such as age, sex, or race that could affect resilience, remain largely unknown. A suggested connection between resolvins and blood pressure control warrants further investigation, especially as it may affect overall CV resilience. Likewise, studies investigating whether therapeutic enhancement of SPM pathways and mediators correlate with improved CVH or CVD outcomes have yet to be performed.

## 4 | ENERGY, ADAPTATION, AND STRESS: MITOCHONDRIAL LINKS FOR CARDIOVASCULAR RESILIENCE, INNATE IMMUNITY, AND COGNITIVE FUNCTION

Mitochondria generate the energy and other signals required for daily functions, response to changes in metabolic demand and other challenges, or recovery from physical insults.^21^ Mitochondrial biology is no different in regard to CVH, especially pertaining to recovery after major events such as myocardial infarction or stroke. Molecular mitochondrial markers have been used as biomarkers after major CV events and to track the state of heart failure for many decades.^22–25^ But how do mitochondria contribute to CVH and resilience? Mitochondria are multifunctional living organelles that perform dozens of functions, such that there is no single measurement that reflects the state of health of mitochondria.^26^ For the purpose of this report, we focused on how the environment influences the state of mitochondria, gene expression, hormonal changes, and how these changes correlate to CVH, resilience, and intersect with other systems. Specifically, much recent focus has been placed on the interdependence between CV and cognitive health.^27,28^ Decreases in either the cellular mitochondrial content or quality control (mitophagy) have been detrimentally implicated in both CVH and aging.

Dysregulated mitochondrial functions in the heart play crucial roles in pathogenesis, leading to decreased energy (ATP production), increased ROS production, and induced apoptosis of cardiomyocytes.^29^ Moreover, distinct categories of CVD are characterized by different profiles of “mitochondrial dysfunction.” Some examples include oxidative stress induced by mitochondrial dysfunction in atherosclerosis, initial increase in the mitochondrial content (i.e., mitochondrial mass) in cardiac hypertrophy followed by a reduction classically linked to abnormal contraction, molecular mitochondrial damage in heart failure leading to increased oxidative stress, and a marked reduction in mitochondrial content may contribute to an exacerbation of heart failure.^30–32^ Recent evidence suggests that mitochondrial content and energy production capacity measured in circulating white blood cells vary dramatically between immune cell types, reflecting unique “mitotypes,”^33^ and mitochondrial content and respiratory chain function may change over days to weeks, suggesting that future research strategies need to allow a more dynamic approach of measuring mitochondrial activity.^33^

Mitochondrial dysregulation is associated not only with poor physical but also with poor mental health.^34^ Depression has been linked to lower mitochondrial content per cell, whereas positive psychological states may increase the capacity of mitochondrial energy production in immune cells,^35^ thereby possibly influencing recovery from a significant event such as myocardial infarction or stroke. This suggests that depression is not only a result of chronic CVD but may also act as a stressor leading to CV decline.^36^ More details regarding allostasis (the process of predictive or anticipatory regulation that enables one to maintain certain vital parameters within healthy limits by changing other parameters),^37,38^ and allostatic load (the process of wear and tear on the body from adapting to chronic stress),^39,40^ will be discussed later under the heading “Psychosocial Aspects of Resilience.” Recently, it has been suggested that mitochondrial functions are not only important for health and recovery but also that mitochondrial DNA (mtDNA) copy number (mtDNAcn, the number of mtDNA copies per cell, often measured in mixed cell populations in blood) may influence whole body metabolism.^41–43^ This suggests that mitochondrial DNA affects gene expression and other factors that affect CVD, such as obesity and dietary responses.^44^ However, mtDNAcn by itself cannot be interpreted and is confounded by several factors, including hematopoiesis.^45^ More studies are needed to elucidate how these processes are linked, particularly across organ systems (e.g., how blood cell’s mitochondrial biology relates to CV function). The role of mitochondrial-nuclear communication, bioenergetics, and metabolism merit particular attention as systemic modulators of tissue resilience across related systems.

The increased interest in the role of mitochondrial Damage Associated Molecular Patterns (DAMPs)-related autoimmunity in the pathogenesis of various diseases has received a recent boost from COVID-19 observational studies. DAMPs are generated through the release of mitochondrial organelle components, including the mtDNA, N-formyl peptides, and cardiolipin, and initiate multiple pro-inflammatory pathways via pattern recognition receptors. Recent data from COVID-19 observational studies showing an association between increased DAMPs levels and higher risk of COVID-19 mortality,^46^ support the increased interest in the importance of mitochondrial DAMPs-related autoimmunity in the pathogenesis of various diseases, including CVD. Additionally, the age of onset and COVID-19 severity were associated with mitochondrial mutations,^47^ suggesting that investigating individual differences in mitochondrial metabolism may provide key understanding of resilience as well as heterogeneity of clinical features associated with COVID-19 infection and its long-term sequelae, including CVD.

Determining the role of mitochondria in CV resilience and well-being hinges on being able to classify individuals who are resilient to specific CVDs. In this regard, the development of methods that could measure mitochondrial variation, stability, and functional phenotypes would provide additional exploratory tools to verify potential connections between mitochondrial health and physiological sensitivity and/or resilience in individuals, and to determine if there are specific mitochondrial markers contributing to CV resilience.

## 5 | AGING AND RESILIENCE

Aging and resilience are interconnected, yet it is crucial to distinguish and elucidate underlying mechanisms for each. Age is the primary risk factor for CVD and other major medical conditions, such as cancer, diabetes, and neurodegenerative diseases. Over the past decade, research has identified nine candidate hallmarks of aging that are grouped into three major categories.^48^ The primary hallmarks cause damage to cellular functions: genomic instability, telomere attrition, epigenetic alterations, and loss of proteostasis. The antagonistic hallmarks are responses to such damage: deregulated nutrient sensing, mitochondrial alterations, and cellular senescence. Finally, the integrative hallmarks are possible culprits of the clinical phenotype: stem cell exhaustion and altered intercellular communication, which ultimately lead to impaired function, organ decline, and death. Each of the aging hallmarks is connected to metabolic alterations.^49^ Thus, understanding the underlying genetic, metabolic, and molecular processes of aging may help identify resilience mechanisms and develop therapeutic interventions that could alleviate, delay, prevent, or even reverse age-related diseases. Understanding of how these hallmarks interplay in resilience would require vast amounts of data and data harmonization in meaningful ways.

Several metabolic-based interventions can increase longevity, including caloric restriction (CR), selective protein and amino acid restriction, and physical exercise. These lifespan-extending maneuvers impose beneficial pleiotropic effects on metabolism. For example, weight reduction by CR results in functional benefits by alleviating the physical complications of obesity, for example, excess intramuscular adipose tissue and joint overload. Beyond the mechanical aspects, CR increases metabolic flexibility and reduces white adipose tissue, particularly visceral fat, which is particularly detrimental for healthy aging due to its pro-inflammatory and diabetogenic activity.^50^ CR extends the lifespan across multiple species.^51^ Two pathways that are significantly linked to CR and life span are the mechanistic target of rapamycin (mTOR) and sirtuin pathways. Both decreasing mTOR activity and increasing sirtuin activity beneficially modulate the health span.

Moreover, CR induces autophagy in human tissues. Autophagy is an intracellular recycling system with multiple anti-aging effects, as it promotes efficient quality control on organelles, supports optimal stem cell activity, improves immunological functions, and inhibits malignant transformation.^52^ Autophagy declines as cells and tissue age. Physical fitness and longevity are also strongly associated, consistent with the common observation that regular exercise reduces morbidity and mortality in humans.^53^ Altogether, the beneficial effects of CR and exercise are implicated in all nine hallmarks of aging. NHLBI and the National Institute of Diabetes and Digestive and Kidney Diseases (NIDDK) recently convened a workshop focusing on mediators of exercise and their connection to health, resilience, and disease.^54^

There is no doubt that a combination of a healthy, anti-inflammatory diet and regular exercise can delay the onset and progression of aging, however, our knowledge of the metabolic and molecular mechanisms involved is still very limited. Insights into the subcellular underpinnings of resilience may result from analyzing the metabolome and transcriptome of healthy aging individuals and CVD patients. Among the main unresolved questions is whether the age-dependent decline in resilience is driven by all, or just a specific subset of the molecular drivers of aging. Similarly, it remains unknown to what degree aging of the CV system compromises tissue/organ resilience, and through this pathway compromises CV and overall health.

## 6 | LESSONS LEARNED: EXCEPTIONAL LONGEVITY AND STROKE RECOVERY WITHOUT COGNITIVE DECLINE SHED LIGHT ON RISK FACTORS FOR CARDIOVASCULAR HEALTH (CVH) AND RESILIENCE

Healthy aging depends largely on a positive interdependence among CVH, cognition, and organ function. Understanding healthy longevity may require a deeper knowledge of CVH throughout the lifespan. Specifically, we need to determine what constitutes healthy aging and what factors contribute to those living with exceptional longevity with no signs of disease. Events that occur early in life, such as childhood obesity, are known to play a major role in CV function and CVD as an individual grows and matures, but their potential connections to CVH and resilience are understudied. The ability to experimentally measure various functional adaptations can provide valuable insights. For instance, determining the factors that enable individuals with exceptional longevity to recover from serious clinical events, for example, stroke recovery with no cognitive decline vs. those who continue to live with increased frailty and inflammation can provide directions for the exploration of mechanisms. Similarly, clarifying the differential impact of risk factors and environmental stress/factors in healthy aging will also be important to integrate the relevant constituents that contribute to CVH and resilience. From a psychological standpoint, the concept of allosteric load is further discussed in the section titled “Psychosocial Aspects of Resilience.”

Healthy life is described as a period of time with no disability (i.e., the ability to perform activities of daily living without difficulty). Incident disability rates are vastly different when comparing age, sex, and race. In fact, age is a strong indicator of survival, disability, and CV events. Though women have higher rates of disability, they still have a longer life expectancy than men.^55–58^ These differences lead to unanswered questions: Can we distinguish between those who are aging vs. those who are dying? Can we identify clues forecasting CV events, and are there patterns of decline that can predict these events?

The adaptive aging measure of recovery may be considered a marker of resilience. To understand adaptive aging, we need to understand the elements that promote diseases such as cancer, dementia, arthritis, osteoporosis, depression, kidney disease, and perceived severity of disease (typically self-rated). The use of the Scale of Aging Vigor in Epidemiology may be useful in assessing frailty.^59^ Older adults who are relatively strong and vigorous tend to live longer. Predicting this decrease in mortality may also depend on assessing measurable outside influences such as socioeconomic status, metabolism, inflammation, and immune risk factors.^60^ Investigating the variations in patients’ ability to recover from a major event such as stroke may lead to clues regarding CV resilience. These types of studies require prospective data prior to and after the event of interest. There has been little focus on these studies due to the lack of prospective data. In one study, Winovich and colleagues^61^ were able to leverage the Cardiovascular Health Study and identify factors that are associated with stroke survival and recovery.^61^ Though disability and cognitive impairment are common outcomes from stroke, much can be learned from those few who do not suffer from these outcomes. Studies/analyses leveraging longitudinal cohorts to identify recovery rates and associated factors could spur hypothesis-driven research and potentially identify modifiable elements that affect recovery. Determining how such factors are influenced by age, sex, and race would further advance the field.

These considerations bring us back to the central question: “What are the key features associated with exceptional (healthy) aging, and does this exceptional aging serve as an indicator of resilience”? One notable study addressing this question leverages the longitudinal Cardiovascular Health Study cohort. Odden et al. focused on gradations of subclinical vascular disease (SVD) burden and devised a way to analyze these categories to determine what allows some participants to live longer with SVD.^62^ Such research highlights questions of which life course factors play into resilience. Do these resilience factors contribute to adaptive aging and offset risk accumulation, resulting in a prolonged health span? Understanding the genetic and environmental factors contributing to healthy aging and recovery from CV events is critical to comprehend resilience.

Existing longitudinal studies offer opportunities to characterize differences between individuals who recover from severe CV events versus those who do not. Moving forward, the identification of stressors and recovery factors associated with CV resilience will shed light on critical areas of this focus. Importantly, multidisciplinary collaborations between biologists, geneticists, epidemiologists, bioinformaticians, social scientists, etc., are needed to develop biological, genetical, and behavioral measures that establish and translate to CV resilience. Additionally, the development and use of metabolome/microbiome assessments during the health span are needed to determine potential genetic signatures of CV and overall resilience.

## 7 | PSYCHOSOCIAL ASPECTS OF RESILIENCE

Psychological resilience may be conceptualized as a predisposition to respond favorably to stressors, as an outcome (i.e., a favorable outcome achieved despite adversity), and/or as a response to stress (i.e., a return to baseline following stress exposure).^63,64^ Each of these conceptualizations have been favorably associated with CV outcomes.^65–67^ The working definition of resilience, “the ability to resist and recover from stress,” is consistent with the latter two conceptualizations. A common element across these is that resilience is best understood in the context of stress exposure. Stress models suggest that achieving psychological resilience depends on the source of the stressor, the controllability of the stressor, the cognitive appraisal of the stressor, the resulting behavioral response to the stressor, and the time course of the stress response.

The Transactional Model of Stress and Coping^68^ and the Allostatic Load model^69^ suggest that resilience may enhance CVH by directly influencing physiological stress responses, or indirectly through influencing adverse health behaviors. In the Transactional Model, resilience may be apparent in one’s perceptions of the stressor (i.e., perceived as not too stressful) and the perception that one has coping resources to manage the stressor. The Allostatic Load model^69^ addresses resilience as a biological adaption to stress. It builds on classic models of biological adaptation to stress (e.g., fight or flight response)^70,71^ with concepts related to the adaptation to stress and the cost of that adaptation (i.e., allostasis and allostatic load). The model also recognizes that perceptions of stress and individual differences in the ability to respond to stress (i.e., epigenetics, environmental resources, past experiences, and behaviors) influence the resulting biological responses.^72–75^ Its central premise is that the chronic activation of physiological systems (i.e., neuroendocrine, CV, immune, and central nervous system), in the presence of perceived stress and the lack of physiological recovery, ultimately causes deterioration in multiple organ systems and increases disease susceptibility.^69^ In this model, resilience has been referred to as successful allostasis, or adaptation to stress, while minimizing the load or “wear and tear” on physiological systems.^76^ Different from other stress models, the Allostatic Load model includes a recovery phase of physiological stress responses. Whether an individual can recover and the duration of such recovery may also be an indicator of resilience consistent with the proposed definition.

Putting these perspectives together suggests that psychological resilience may be best understood and measured as an adaptive physiological response trajectory among other possible trajectories that may follow exposure to stress rather than as a steady-state.^77,78^ The critical component is the pattern of an individual’s initial response to a stressful event and how the response returns to a pre-stress baseline. In addition to a resilience trajectory, other possible trajectories include resistance, recovery, relapsing/remitting, delayed dysfunction, and chronic dysfunction. Additionally, resilience encompasses many other aspects, such as the abilities and resources available that enable an individual to respond favorably to stressors, thereby promoting better CVH outcomes through these processes.

In measuring and understanding psychological resilience in CVH, important considerations include the: (1) source of stressor, (2) stress perception (i.e., severity and controllability), (3) coping responses, and (4) time course of stress adaptation (i.e., physiologically, psychologically, and behaviorally). An additional layer of complexity is whether an individual may be resilient to different stressors at different time points. Short-term research efforts to build evidence in these areas include studies using ecological momentary assessments of stress and response as well as just-in-time adaptive resilience interventions. An example of more long-term research efforts includes utilizing information emerging from the Human Resilience Project,^79^ designed to collect and integrate genetic data to better understand individual variability in resilience.

Mechanisms of psychological resilience have particular relevance to primordial risk, resilience, and recovery in CVD. The Transactional and Allostatic Load models are frameworks employed to explain how the response to perceived stress contributes to resilience.

Studying the relationship between psychological resilience and CVH will require resilience assessments over time, including age-dependent resilience analyses and adoption of a life-course approach. Data and measures of resilience also need to be integrated from the cellular, tissue, individual, and eventually to the population level. Introduction of interventions into studies allows investigations of resilience as a dynamic process. Existing clinical cohorts offer opportunities to accommodate different assessments and indicators of resilience (e.g., cognitive function, CVH, microbiome, etc.). A systems approach should be employed for measurements of allostatic load and its changes over time (i.e., trajectories) in response to defined stressors to characterize the determinants that confer resilience, which could lead to the development of a ‘resiliome.’

The COVID-19 worldwide pandemic has offered the unfortunate opportunity to examine psychological resilience in response to a natural experiment of stressor exposure. A rapid review and meta-analysis were conducted of longitudinal studies and natural experiments of the association of COVID-19 lockdown requirements and psychological health and resilience.^80^

This review identified 25 studies with 72 004 participants and discovered that lockdown requirements overall had small effects on psychological health symptoms but substantial heterogeneity. The authors concluded that this natural experiment of stressor exposure did not have uniform exacerbating effects, as some were psychologically resilient to this stressor exposure. These data will need to be updated as COVID-19 and its sequelae continue to unfold.

## 8 | RACE, RISK, AND RESILIENCE

Resilience is evinced in the occurrence of adversities presented to the individual or biological system. Substantial research demonstrates that African Americans (AAs) have a higher risk of developing CVD than other racial groups, even after adjusting for socioeconomic status (SES) and traditional risk factors. While cataloging these clear group disparities represents a critical step toward addressing health inequities, a focus only on group comparisons can present a missed opportunity to evaluate evidence of resilience in the face of CV stressors among AAs, obscuring within-group successes. Investigating variation in health within groups that traditionally are considered to have worse health outcomes (such as AAs or Hispanics) allows an emphasis on those who remained well under the extreme conditions that otherwise produce large-scale disparities. For example, while the Jackson Heart Study reports the prevalence of hypertension among AA men over 65 years old to be greater than 80%, little attention is given to the 20% who maintain normal blood pressures in later life despite facing similar SES, environmental, biological, and psychosocial exposures.^81^ Understanding the individual and environmental contributors to variation in health and disease among AAs may give insights into the nature of physiological resilience.

Contextual factors (external to the person or biological system) can serve to erode resilience, exacerbating the impact of risk factors and impairing recovery from internal or environmental stressors. Conversely, context can promote resilience in the face of individual or collective risk. For instance, different neighborhoods in the Atlanta metropolitan area inhabited by AAs of similar socioeconomic status have been shown to have vastly different CVH statistics.^82^ Studying variation among neighborhoods within a region and ethnic group known for poor health outcomes may reveal mini-“blue zones” with unexpectedly good CVH. Characteristics of the inhabitants, the nature of their social interactions, and aspects of the physical environment may individually or jointly impact resilience manifested by lower event rates and the prevalence of CVD. Further, inhabitants’ biomarkers and risk factor profiles may yield information about how contextual factors transduce into biological resilience.

Thus, cities such as Atlanta can be a perfect ‘laboratory’ due to their inherent wide variation in SES within racial groups, and their large and dense heterogeneous populations that reside near diverse urban, suburban, and rural communities,^83^ yet not much epidemiological research has been performed in these richly heterogeneous zones. The Morehouse Emory Cardiovascular Center for Health Equity (MECA) project is establishing a collaborative, multidisciplinary resilience research platform in the Atlanta Metropolitan area - home to 2 million AA residents. “Resilient” and “at risk” neighborhoods are identified across Metro Atlanta to allow a multilayered comparison of the occurrence of CVD hospitalizations, emergency room visits, and deaths among this high-risk Southern Black population. Early studies led by MECA’s population science group show a strong association of neighborhood residents’ individual resilience characteristics and neighborhood group CVD outcomes (i.e., individuals with a psychosocially “resilient profile” tend to populate neighborhoods with unexpectedly low rates of neighborhood CV death, CVD emergency room visits and CVD hospitalizations).^84^ The relationship between resilient and risk factors and Life’s Simple 7 (LS7), a measure of individual CVH^85–87^ are examined. Different perceptions of the external environment such as esthetic quality, violence, safety, and healthy food access are being studied. In addition, different psychological factors, sleep, and social stressors are being identified. Preliminary results indicate that both neighborhood and individual factors impact on resilience outcomes. This basic science project also investigates various metabolites and transcriptomes and their relationship to resilience. A complementary MECA analysis led by a team of social and clinical scientists affirmed that the prevalence of ideal scores on LS7 was associated independently with both contextual (physical and social) characteristics of the participant’s neighborhood and her/his individual psychological attributes, such as purpose in life, environmental mastery, and optimism.^82^ Taken together, these population and individual-level analyses suggest that certain environmental and individual features work independently to produce the vastly different neighborhood outcomes and individual CVH profiles within a population presumed to be at uniformly high risk.

Further clinical, laboratory, and psychological assessments, including biomarker assays (e.g., cytokines, oxidative stress markers), measures of vascular function (arterial stiffness, endothelial cell function studies), and assessments of regenerative capacity (progenitor cell populations) are underway to further characterize individuals who have unexpectedly high (or low) levels of CVH. MECA will also employ multi-omics methodology to decipher epigenetic and metabolomic profiles of individuals meeting study criteria for CV resilience (and risk), before and after lifestyle interventions. These exploratory studies will generate at least as many hypotheses as conclusions, and ultimately, larger sample sizes than the original cohort will be needed to strengthen and expand initial work. However, even now, these studies serve a critical function as the field of resilience evolves and embraces the question of survival and health of humans facing large-scale, chronic stressors embedded in both the physical and psychosocial milieu—a state of chronic stress occasionally exacerbated by acute group trauma (e.g., George Floyd murder and related occurrences) or disaster (e.g., a pandemic such as COVID-19, with disproportionate levels of infection and death).

Research in the area of race and resilience offers many opportunities along with challenges. A comprehensive understanding of resilience will require integrative teams of multiple disciplines, which offer unique advantages but also pose the inherent difficulties associated with cross-disciplinary communication and collaboration. Basic definitions and conceptual frameworks still require refinement to guide the field. The dynamic nature of resilience and stress leads to challenges in identifying acute versus chronic stressors and assessing dynamic processes at a single time point. Opportunities abound for assessing resilience across the lifespan and observing at the tails of the distribution for those who are truly robust in their health and well-being. There is a need to develop “stress tests” for the resilience of key CVH systems and for the investigation of key CV mechanisms of modifiers of resilience, such as sleep and inflammation at the cellular and organismal level. Inclusion of population-based studies of AA and Native American populations who have survived historical racist and genocidal stressors may offer critical social, psychological, behavioral, epigenetic, and multi-omic insights into the phenomenon of human resilience.

Racial disparities in CVD (ranging from primordial risk to premature mortality) have been well-documented and analyzed mainly from a perspective of vulnerability and poor outcomes among AAs. More recently, the racial disparities in COVID-19 infection, hospitalization, and death have been documented in minority populations.^88,89^ While this evidence is critically important, it may reflect an unbalanced perspective that looks past within group heterogeneity and signals of resilience and vigor in the face of severe, transgenerational stressors. While excess risk is undeniable among this and other distinct groups, survival amid multilayered and protracted stressors may also reveal compelling instances of resilience in AA and other American populations who have survived in the face of near genocidal pressures (e.g., American Indian/Alaska Natives). The Jackson Heart Study, the Strong Heart Study, and others not only address the lack of within-group assessment of CVH but also provide a seminal opportunity to study the combination of environmental and individual contributors to physiological CV resilience, as well as resilience to other diseases, on a longitudinal basis.

Studying the relationships between Race, Risk, and Resilience and CVH should leverage existing AA and Indigenous peoples’ cohorts to accommodate assessments and indicators of resilience (e.g., cognitive function, CVH, microbiome, etc.). Existent data and biospecimen repositories need to be mined for evidence of health despite substantial social, psychological, environmental, and biological stressors over time. New, ‘next generation’ racially diverse cohorts need to be established by taking advantage of both classic epidemiology and high technological assessments of the “exposome,” including remote monitoring of the environment, activities and studies using ecological momentary assessments (minimizing participant burden and research costs related to “in-clinic” phenotyping) are needed, integrating data across scales and life span.

## 9 | SUMMARY OF RESEARCH OPPORTUNITIES

Resilience is a term in broad use that is readily understood, yet requires clarification, especially when referring to research on processes, traits, stressors, and outcomes that are multilayered, multifaceted, and mutable over the life course. We have worked with multidisciplinary experts to create a shared understanding and a working definition related to CV resilience. A broad working group with representatives from several Institutes within the National Institutes of Health (NIH) has also been at work adopting a definition to advance the science of resilience across many domains enabled by various NIH Institutes.^90^ A potential resilience research tool to be considered when designing studies intending to test resilience was also developed and proposed by this NIH working group.^91^

The ability to resist and recover from stress is necessary because threats to biological systems’ integrity and/or viability, from nucleotides to neighborhoods, are unavoidable, routine, and recur throughout the lifespan. A rigorous exploration of the underpinnings of resilience is a demanding and exciting direction for future heart, lung, blood, and sleep research and beyond. Understanding internal (biological and psychological) and contextual (physical and social environmental) factors that promote health in the face of adverse conditions represents a promising frontier that may yield new preventive and therapeutic interventions. In addition, further research on risk and resilience to SARS-CoV-2 infection, the development of severe illness and post-acute sequelae of COVID-19 are needed, as this pandemic is an exemplary model that involves almost all of the resilience aspects discussed herein.

Specifically, enhancing CV resilience will promote CVH, prevent CVD, improve recovery from CV morbidity, and help extend the overall health- and lifespan. We propose several scientific opportunities that could help reduce the current significant gaps in our understanding of the multi-faceted nature of CV and overall resilience.

## Data Availability

Data sharing is not applicable to this article as no datasets were generated or analyzed for this current report.

## Abbreviations:

AA: African American
COVID-19: coronavirus disease 2019
CR: caloric restriction
CV: cardiovascular
CVD: cardiovascular disease
CVH: cardiovascular health
EMR: electronic medical record
EPA: eicosapentaenoic acid
GWAS: genome-wide association studies
LS7: Life’s Simple 7
MECA: Morehouse Emory Cardiovascular Center for Health Equity
mtDNA: mitochondrial DNA
mtDNAcn: mtDNA copy number
mTOR: mechanistic target of rapamycin
NHLBI: National Heart, Lung, and Blood Institute
NIDDK: National Institute of Diabetes and Digestive and Kidney Diseases
PCSK9: proprotein convertase subtilisin/kexin type 9
PUFAs: polyunsaturated fatty acids
ROS: reactive oxygen species
SARS-CoV-2: severe acute respiratory syndrome coronavirus 2
SES: socioeconomic status
SVD: subclinical vascular disease
VEGF: Vascular endothelial growth factor
